# Corrigendum: Tick innate immune responses to hematophagy and *Ehrlichia* infection at single-cell resolution

**DOI:** 10.3389/fimmu.2024.1523328

**Published:** 2024-11-26

**Authors:** Abdulsalam Adegoke, Jose M. C. Ribeiro, Ryan C. Smith, Shahid Karim

**Affiliations:** ^1^ School of Biological, Environmental, and Earth Sciences, The University of Southern Mississippi, Hattiesburg, MS, United States; ^2^ Vector Biology Section, Laboratory of Malaria and Vector Research, National Institute of Allergy and Infectious Diseases, National Institutes of Health, Rockville, MD, United States; ^3^ Department of Plant Pathology, Entomology, and Microbiology, Iowa State University, Ames, IA, United States

**Keywords:** hemocytes, 10X Genomics, *Amblyomma americanum*, *Ehrlichia chaffeensis*, single cell, RNA-sequencing

In the published article, there was an error in the **Data availability statement**. The wrong accession number was given in the **Data availability statement**: “The raw datasets generated in this study are deposited in the NCBI Transcriptome Shotgun Annotation (TSA) repository under the accession number PRJNA226980.”

The correct **Data availability statement** appears below.

“The raw datasets generated in this study are deposited in the NCBI Transcriptome Shotgun Annotation (TSA) repository under the accession number PRJNA1017904 (https://www.ncbi.nlm.nih.gov/bioproject/?term=PRJNA1017904).”

The authors apologize for this error and state that this does not change the scientific conclusions of the article in any way. The original article has been updated.

