# Supplementary figures and images for: Tick innate immune responses to hematophagy and Ehrlichia infection at single-cell resolution
Source: Front Immunol. 2024 Jan 11;14:1305976. doi: 10.3389/fimmu.2023.1305976 (PMC10808623; doi:10.3389/fimmu.2023.1305976)

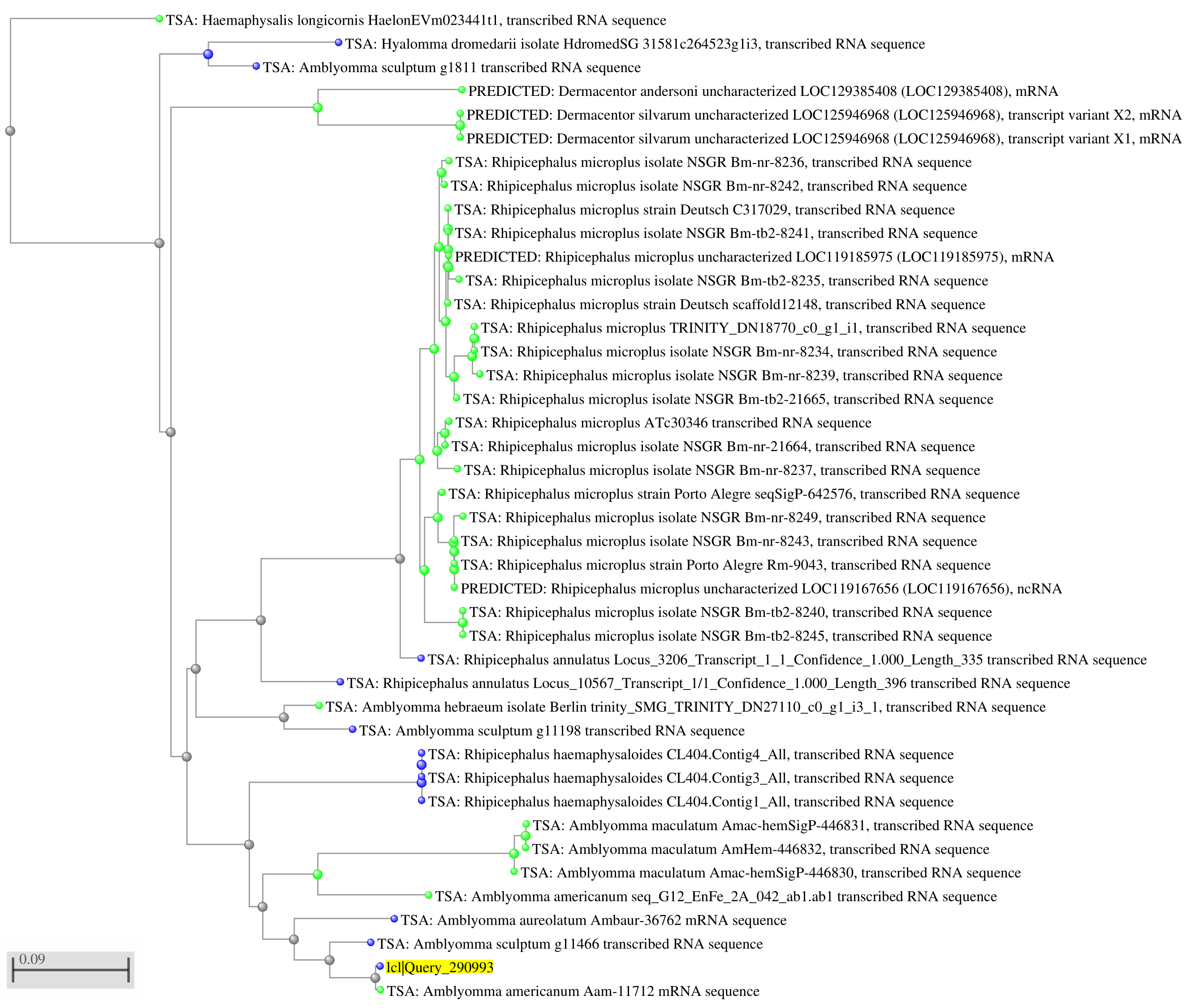

Supplement: Supplementary Figure 1 — The neighbor-joining phylogenetic tree from the aligned lipocalin sequences following 1000 bootstraps. The bar at the bottom represents 20% amino acid diversity. The numbers at the nodes indicate the percentage bootstrap support. [file Image_1.tiff]

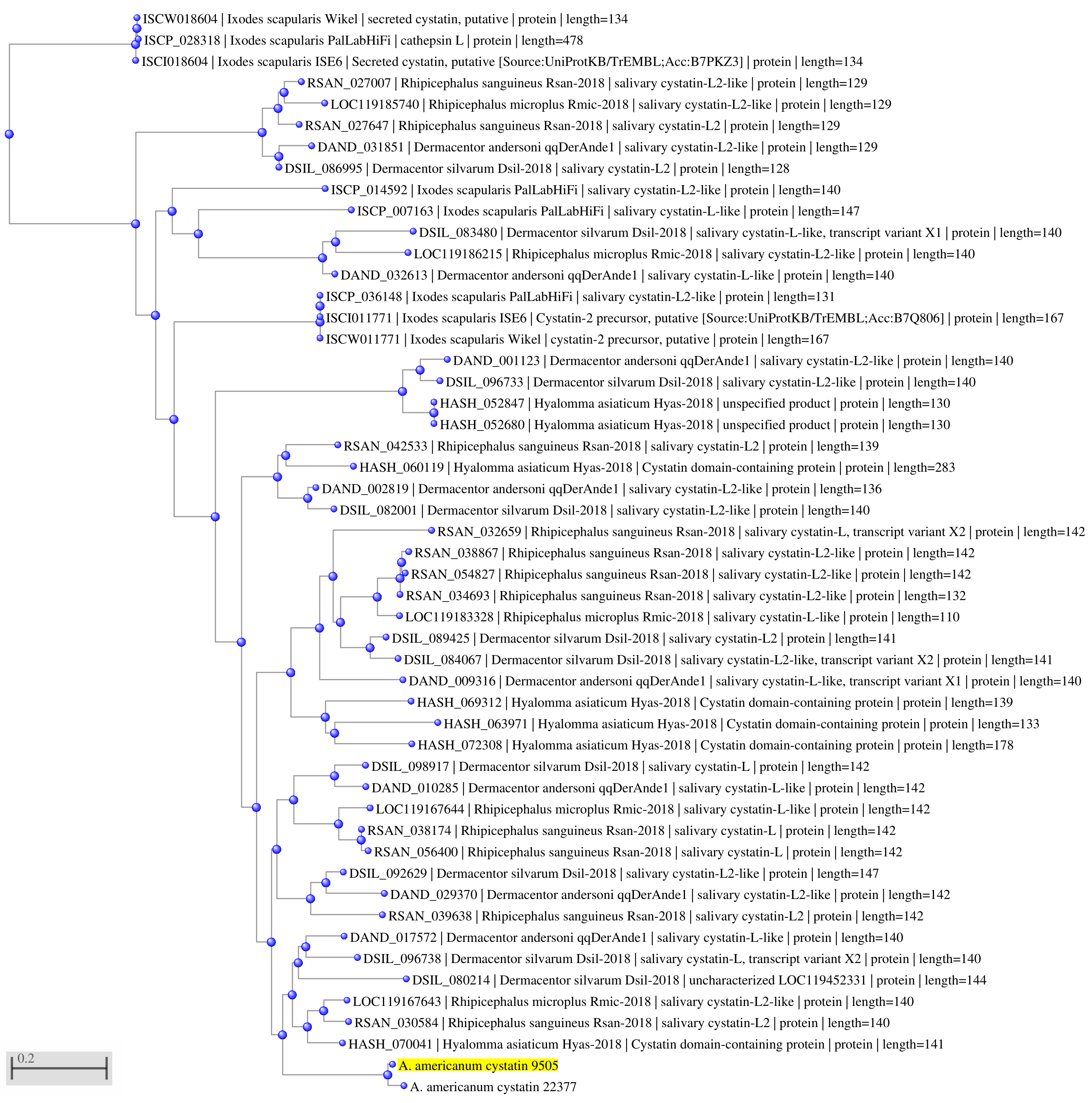

Supplement: Supplementary Figure 2 — The neighbor-joining phylogenetic tree from the aligned cystatin sequences following 1000 bootstraps. The bar at the bottom represents 20% amino acid diversity. The numbers at the nodes indicate the percentage bootstrap support. [file Image_2.tiff]

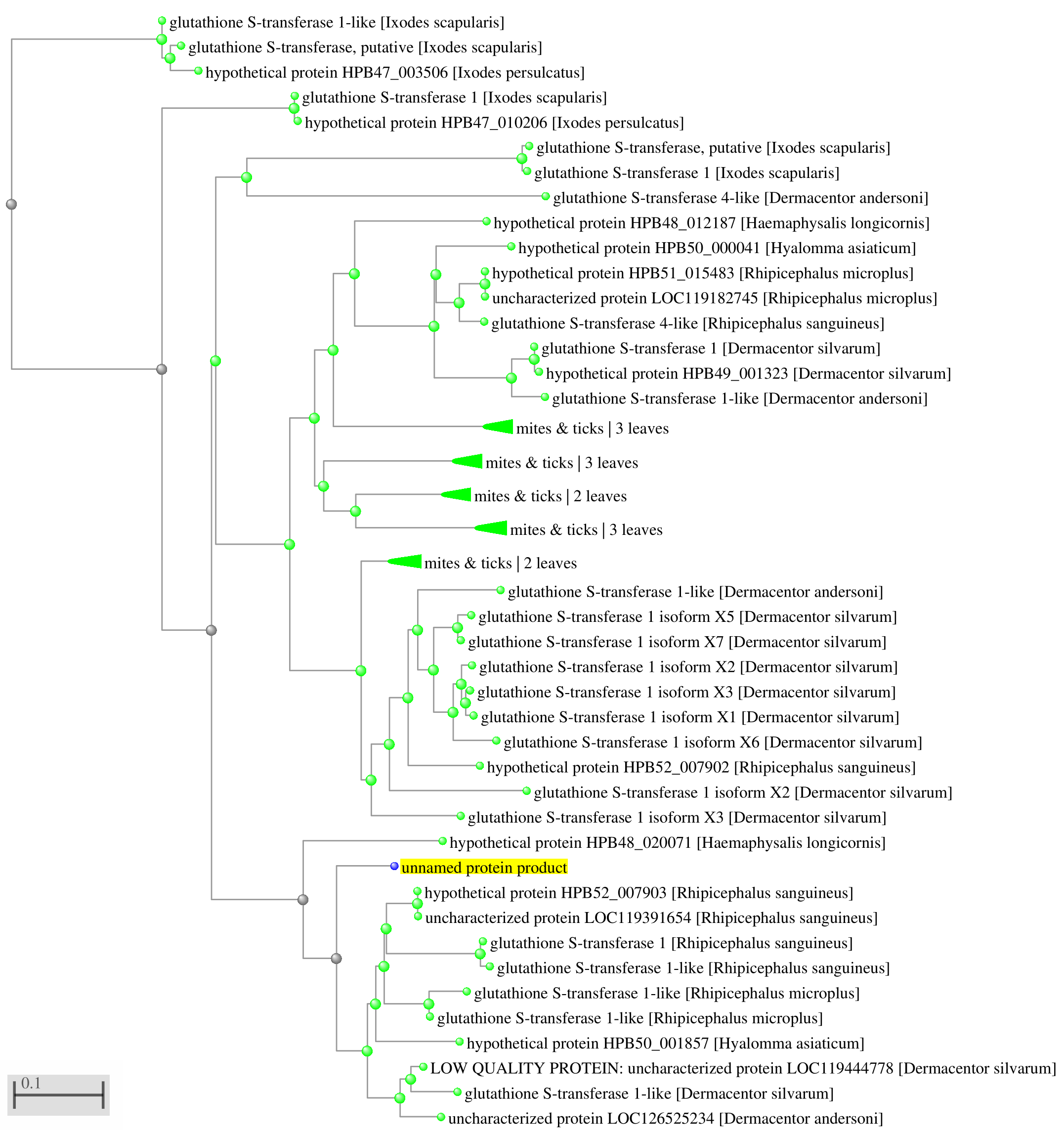

Supplement: Supplementary Figure 3 — The neighbor-joining phylogenetic tree from the aligned glutathione s-transferase sequences following 1000 bootstraps. The bar at the bottom represents 20% amino acid diversity. The numbers at the nodes indicate the percentage bootstrap support. [file Image_3.tiff]
